# Off-Target MAP2K7 Inhibition Drives the Antileukemic Activity of the PD-1/PD-L1 inhibitor ACS-69 in Pediatric T-ALL

**DOI:** 10.21203/rs.3.rs-10474945/v1

**Published:** 2026-08-14

**Authors:** Eric J. Malmberg, Cory Seth Bridges, Saptarsi Shai, Evan Beatty, Gina M. Connors, Monica Puppi, Karl A. Scheidt, H. Daniel Lacorazza

**Affiliations:** Baylor College of Medicine; Baylor College of Medicine; Baylor College of Medicine; Baylor College of Medicine, Texas Children’s Hospital; Northwestern University; Baylor College of Medicine; Northwestern University; Baylor College of Medicine

**Keywords:** T-ALL, MAP2K7, ASC69, kinase inhibitor

## Abstract

T-cell acute lymphoblastic leukemia (T-ALL) remains a high-risk pediatric malignancy characterized by aggressive disease behavior and poor outcomes after relapse, underscoring the need for novel targeted therapies. Our group reported that aberrant activation of the MAP2K7–JNK signaling axis is implicated in T-ALL pathogenesis, promoting leukemic cell survival, proliferation, and treatment resistance. In this study, we evaluated the anti-leukemic properties of the compound ASC69 in T-ALL cell lines and patient-derived xenograft cells using cell viability, apoptosis, and cell cycle analyses. Pathway inhibition was examined by immunoblotting. ASC69 exhibited dose-dependent cytotoxicity across multiple T-ALL cell lines, with IC_50_ values in the low nanomolar range, while showing reduced cytotoxicity against normal human bone marrow cells. Genetic ablation of the MAP2K7 gene in Jurkat cells significantly increased the ASC69 IC_50_, supporting MAP2K7 as the primary functional target. Mechanistically, ASC69 induced G2/M cell-cycle arrest and apoptosis, as determined by Annexin V staining and cleavage of PARP and Caspase-3. *In vitro* kinase assays demonstrated direct inhibition of MAP2K7 enzymatic activity across multiple activation conformations, including wild-type, an active phosphomimetic mutant, and an active phosphomimetic mutant with a 75-deletion in the N-terminus. Consistent with on-target pathway engagement, ASC69 inhibited both basal and stress-induced phosphorylation of JNK and ATF2 in T-ALL cell lines. Importantly, ASC69 demonstrated cytotoxic activity in T-ALL patient-derived xenograft cells and synergized with standard-of-care T-ALL drugs. Collectively, these findings establish ASC69 as a promising MAP2K7-targeted therapeutic candidate and support preclinical studies in T-ALL models.

## Introduction

Leukemia is the most common childhood cancer, accounting for approximately 30% of all malignancies in children under 15 years of age. Acute lymphoblastic leukemia (ALL) accounts for 80% of these cases, of which 12–15% are T-cell ALL (T-ALL) [1–5]. T-ALL presents significant clinical challenges due to its aggressive behavior, frequent extramedullary involvement (e.g., CNS in 5–10% of cases at diagnosis), and a higher risk of treatment failure than B-cell ALL (B-ALL) [6, 7]. Current treatment regimens involve intensive multi-agent chemotherapy, including vincristine, corticosteroids, and asparaginase; consolidation with high-dose methotrexate and cytarabine; and maintenance with mercaptopurine and methotrexate [5, 8]. These regimens have improved outcomes, achieving 5-year event-free survival (EFS) rates of over 85% in children [6, 7, 9]. However, relapse rates remain high (15–20%), and relapsed disease carries a poor prognosis with less than 25% EFS, making it a major contributor to cancer-related mortality in children and underscoring the need for novel therapeutic strategies [7, 10, 11].

Our group reported that the reprogramming factor Krüppel-like factor 4 (KLF4) has tumor-suppressive function in pediatric T-ALL [12–15]. Loss of KLF4 accelerated NOTCH1-induced T-ALL in a mouse model, associated with increased proliferation, expansion of leukemia-initiating cells (LICs), and upregulation of mitogen-activated protein kinase 7 (MAP2K7) expression compared to healthy cells, leading to hyperactivation of the JNK stress signaling pathway [12, 15–17]. MAP2K7 is a dual-specificity kinase that selectively phosphorylates JNK isoforms, distinguishing it from other kinases, such as MAP2K4, which activates both JNK and p38 MAPKs [16, 18–21]. Similar to the mouse model, lymphoblasts from children with T-ALL showed epigenetic silencing of the KLF4 gene, which was associated with upregulation and hyperactivation of MAP2K7, thereby driving phosphorylation of downstream effectors such as ATF2 and c-JUN, contributing to leukemic proliferation, cell cycle progression, LIC expansion, and chemotherapy resistance [12, 22, 23]. In contrast, MAP2K7 acts as a negative regulator of proliferation in normal hematopoietic stem and progenitor cells, where its loss leads to hyperproliferation, illustrating a context-dependent role [18]. MAP2K7 plays a role in other hematological malignancies, but lacks the distinctive KLF4–MAP2K7–JNK axis observed in T-ALL [16, 24]. Because MAP2K7 selectively drives oncogenic JNK signaling in leukemic cells, its inhibition represents a compelling therapeutic strategy for eradicating LICs and inhibiting leukemia burden [16, 23].

ASC69 (also known as APY69) is a small-molecule ligand identified through structural and screening efforts as a PD/PD-L1 inhibitor [25]. ASC69 belongs to the 4-aminopyrazolylpyrimidines family and can compete with ATP for hinge binding, thereby acting as a kinase inhibitor. Schröder and colleagues reported that ASC69 can inhibit MAP2K7, with an IC_50_ of 0.8 nM, a finding confirmed by analysis of the co-crystal structure [26, 27]. In this study, we investigated whether ASC69 induces cytotoxicity by inhibiting MAP2K7 and suppressing downstream JNK signaling in pediatric T-ALL. Our results demonstrate that ASC69 inhibits MAP2K7 enzymatic activity *in vitro*, reduces JNK phosphorylation, decreases leukemic cell proliferation, causes cell cycle arrest, and induces apoptosis in a panel of T-ALL cell lines. These effects align with published work showing that pharmacologic inhibition of MAP2K7, including probes targeting the kinase active site, decreases proliferation in T-ALL cell lines and clinical samples [16, 23, 28]. Collectively, these findings suggest that ASC69-mediated inhibition of MAP2K7 holds promise as a targeted therapeutic strategy in T-ALL.

## Methods

### T-ALL cell inhibition.

T-ALL cell lines RMPI-8402, DND-41, Ichikawa, Jurkat, CCRF-CEM, Molt-3, KOPT-K1, and ALL-SIL were cultured in RPMI media (VWR) supplemented with 10% fetal bovine serum (FBS Gemini), 1% penicillin and streptomycin (ThermoFisher), and 1% Glutamax (ThermoFisher). Cells were maintained under 5% CO_2_ at 37°C. Cell growth was monitored, and all experiments were performed during the logarithmic growth phase. Cells were tested for mycoplasma and authenticated using short tandem repeat fingerprinting every 6 months. ASC69 (Medchem Express) was diluted to a stock concentration of 10 mM in DMSO and further diluted in RPMI media prior to addition to cell cultures.

### Patient-Derived Xenograft (PDX).

Bone marrow samples were previously collected at diagnosis from patients with T-ALL at the Texas Children’s Cancer and Hematology Center[28]. Samples were collected after written informed consent was obtained from all patients under a research protocol approved by the institutional review board, and deidentified samples were provided to our group. Cells were transplanted into NSG mice, which were monitored for disease progression. Human leukemic blasts (PDX cells) were collected and cryopreserved. Prior to *in vitro* experiments, cells were thawed, placed in pre-warmed X-vivo serum-free media (Lonza) for 3–4 hours, and counted using a Bio-Rad TC20 automated cell counter with Trypan Blue to determine cell viability. Cell viability, Annexin V binding assays, and immunoblots were performed 24 hours after ASC69 treatment.

### Immunoblots.

Cells were lysed in Blue Loading Buffer Pack (Cell Signaling Technology) supplemented with 5% v/v β-mercaptoethanol (Thermofisher), 1 mM PMSF (Sigma), and 1× protease inhibitor cocktail (ThermoFisher). Protein lysates were sonicated to shear DNA, heated at 95°C for 5 min prior to loading, and loaded onto pre-cast gels (Invitrogen) and run at 125 V for 60 min. Proteins were transferred to PVDF membranes (ThermoFisher) using the ThermoFisher iBlot 2 transfer device with Program 0 (20 V for 1 min, 24 V for 4 min, 25 V for 2 min). Membranes were blocked with phosphate-buffered saline (VWR) with 0.1% Tween-20 (Millipore Sigma) containing 5% nonfat dry milk (Bio-Rad) for 1 hour at room temperature. Primary antibodies used were purchased from Cell Signaling Technology and include: phospho-SAPK/JNK, SAPK/JNK, phospho-ATF-2, ATF-2, phospho-P38, p38, cleaved PARP, and cleaved caspase-3. The secondary antibody used was an anti-rabbit HRP-linked IgG, diluted 1:10,000. For actin, we used Direct-Blot^™^ HRP anti-β-actin antibody (BioLegend) diluted 1:10,000 and incubated at room temperature for 30 min. Primary antibodies were incubated in PBST with 5% blocker overnight at 4°C at a 1:1000 dilution. The next day, membranes were washed three times in PBST and incubated with secondary antibodies for 1 hour at room temperature. Horseradish peroxidase (HRP) signal was visualized using SuperSignal^™^ West Femto Maximum Sensitivity Substrate (ThermoFisher Scientific). To visualize phosphorylated proteins, membranes were blocked in Tris-buffered Saline (50 mM Tris-Base, 150 mM sodium chloride, pH 7.4) containing 0.1% Tween-20 and 3 w/v BSA (Millipore Sigma). Blots were imaged using the iBright 1500 imaging system (ThermoFisher). Membranes were stripped with Restore^™^ PLUS Western Blot Stripping Buffer (ThermoFisher) for 5–10 min at room temperature, then re-blocked for 1 h at room temperature in PBST with 5% blotting-grade blocker before probing with a new primary antibody.

### Sorbitol-Induced MAP2K7 Activation.

To induce metabolic activation of MAP2K7, cells (1 × 10^6^ cells) were treated with vehicle (control) or 400 mM sorbitol (Sigma-Aldrich) for 3–4 hours at 37°C with 5% CO_2_. Where indicated, ASC69 was added to a final concentration of 5 μM, and cells were incubated for an additional 4 hours. Following treatment, cells were collected, washed with PBS, and lysed as described above for immunoblot analysis.

### Cell Viability Assay.

Cells were plated in white 96-well plates (20,000 cells/well), and serial dilutions of ASC69 were added to reach a total volume of 100 μl/well. Plates were incubated for 48 hours at 37°C with 5% CO_2_. Cell viability was measured using the CellTiter-Glo 2.0 Luminescent Cell Viability Assay (Promega). Briefly, 100 μL of CellTiter-Glo 2.0 Reagent was added to each well, and plates were incubated with shaking at 25°C for 10 minutes before measuring luminescence using a Luminoskan luminometer.

### MAP2K7 Kinase Assay.

Human full-length MAP2K7, phosphomimetic MAP2K7 S271D/T275D, and MAP2K7 S271D/T275D del75 were obtained from Sinobiological (0.5 mg at 0.79 mg/mL). Kinase-dead JNK2 (100 μg at 0.36 mg/mL, ThermoFisher), 10 mM ATP stock (diluted to 200 μM), and test compound ASC69 were used. Kinase buffer was prepared by diluting 5× stock with water and adding 0.25 mM DTT. T-ALL cell lines were plated in white 96-well plates, with 320 nM MAP2K7 and 345 nM JNK2 added, then completed with 7.5 μL of kinase buffer. Controls included JNK2 with kinase buffer or 5 μL of 10 μM drug and 7.5 μL kinase buffer. Next, 5 μL of ASC69 was added, and plates were pre-incubated for 30 minutes at 24°C. The reaction was initiated by adding 12.5 μL of 200 μM ATP and incubated for 60 minutes at 24°C in the dark using an Eppendorf Thermomixer C with the 96-well plate attachment and lid. Then, 25 μL of ADP-Glo^™^ Reagent was added and incubated for 40 minutes at 24°C, followed by 50 μL of Kinase Detection Reagent, which was incubated for 30 minutes at 24°C. Luminescence was measured using a Luminoskan luminometer.

### Apoptosis Assay.

Apoptosis was evaluated by flow cytometry using PE Annexin V (BD Pharmingen). TALL cells were treated with ASC69 for 24 hours, washed, and resuspended in Annexin V Binding Buffer (10 mM HEPES, pH 7.4, 140 mM NaCl, 2.5 mM CaCl_2_) at a concentration of 1 × 10^6^ cells/mL. Cells (1 × 10^5^ cells) were incubated with PE Annexin V for 15 minutes at room temperature (25°C). Subsequently, 400 μL of binding buffer was added, and samples were analyzed by flow cytometry within one hour.

### Cell Cycle Analysis.

For DNA content analysis, T-ALL cells were treated with ASC69 (2× IC50) for 24 hours. Cells were harvested from suspension cultures (1×10^6^), washed with PBS, and incubated with a solution containing 1 mg/mL propidium iodide (Sigma-Aldrich), 0.1% sodium citrate, 0.1% Triton X-100, and 2 mg/mL RNase A (Invitrogen) for 30 minutes at room temperature. Samples were then analyzed by flow cytometry to determine DNA content, and DNA analysis was performed in FloJo software, gating on singlets (excluding sub-G1 apoptotic cells). To assess cell proliferation and DNA content, we used the Click-ItTM Plus EdU Flow Cytometry Assay Kit (ThermoFisher Scientific). T-ALL cell lines were treated with vehicle (DMSO) or ASC69 (2× IC50) for 24 hours. Cells were pulsed with 5-ethynyl-2′-deoxyuridine (EdU), resuspended in staining buffer, and stained with propidium iodide (50 μg/ml) solution containing RNase A (200 μg/ml) to evaluate DNA content. Samples were incubated at room temperature in the dark for 15–20 minutes before analysis. Flow cytometry was performed to simultaneously measure EdU incorporation and DNA content. Data were collected on a flow cytometer and analyzed using appropriate gating strategies to identify different cell cycle phases.

### Drug Combination Studies.

The effect of combining ASC69 with therapeutic agents was evaluated by co-treating with dexamethasone, asparaginase, vincristine, nelarabine, and cytarabine (Medchem Express). T-ALL cells (20,000 cells/50 μl/well) were plated in white 96-well plates, and 50 μl of drug dilutions was added to the wells, bringing the final volume to 100 μl. Plates were incubated for 48 hours at 37°C with 5% CO_2_. Cell viability was measured as described above. Drug combination response data were imported into R (v4.5.3) and analyzed using the SynergyFinder package (v3.18.0) [29]. Pairwise drug interaction scores were computed using the highest single-agent (HSA) reference model and visualized as three-dimensional dose-response surface plots, with corresponding two-dimensional contour-level plots.

### Statistical Analysis.

Statistical significance among experimental groups in apoptosis assays was determined using a one-way analysis of variance (ANOVA). Pairwise drug interaction scores were calculated using the highest single agent (HSA) reference model.

## Results

### ASC69 induces Apoptosis and G2/M cell cycle arrest in T-ALL Cell lines.

To assess the cytotoxicity of ASC69 in T-ALL, we conducted cell viability assays on a panel of T-ALL cell lines. Cell viability was measured by ATP production after 48 hours of treatment with different concentrations of ASC69. ASC69 showed dose-dependent cytotoxic effects in T-ALL cell lines, with IC_50_ values in the low-nanomolar range (5–40 nM), using a lymphoblastoid cell line (LCL) as a non-leukemic control (Fig. 1 and Table 1). LCL cells are derived from primary human B cells, immortalized using the Epstein-Barr virus, and used as a human lymphoid baseline.

To elucidate the mechanism of cytotoxicity, we investigated whether ASC69 induces apoptosis. T-ALL cell lines were treated with either vehicle or ASC69 at different concentrations, stained with Annexin V, and analyzed by flow cytometry. ASC69 induced a dose-dependent increase in Annexin V-positive cells in most T-ALL cell lines compared to vehicle-treated controls (Fig. 2a). Apoptosis was further validated by increased cleavage of PARP and caspase-3, two proteolytic events of apoptosis (Fig. 2b). T-ALL cell lines with higher annexin V binding also showed increased levels of both cleaved PARP and cleaved caspase-3. Together, these findings indicate that apoptosis is a primary mechanism of ASC69-induced cytotoxicity in T-ALL.

To further investigate the mechanism of cytotoxicity, we examined the effects of ASC69 on cell cycle progression and analyzed DNA content, gating on singlets and excluding sub-G1 cells. ASC69 induced a significant G2/M cell cycle arrest in RPMI-8402 and Jurkat cells (Fig. 3a). This G2/M arrest was observed in most T-ALL cell lines used in this study (Fig. 3b). LCL cells exhibited an increase in the G1 phase following ASC69 treatment (Fig. 3b). To gain further mechanistic insight and more accurately differentiate actively proliferating cells (S-phase) from cells with 2n DNA content (G2/M), treated cells were analyzed using EdU incorporation and DNA content. Notably, a higher percentage of T-ALL cells accumulated in the G2/M phase following ASC69 treatment (Fig. 3c). This indicates that the observed increase in G2/M reflects an arrest rather than changes in S-phase progression or incomplete DNA replication. Together, these data demonstrate that ASC69 induces cytotoxicity in T-ALL cells through a G2/M cell cycle arrest and apoptosis.

### ASC69 cytotoxicity is mediated through MAP2K7 inhibition

To determine whether ASC69 directly inhibits MAP2K7 catalytic activity, we performed a biochemical kinase assay using recombinant human MAP2K7. Kinase activity was assessed for wild-type MAP2K7, a phosphomimetic mutant (S271D/T275D), and the phosphomimetic mutant lacking 75 amino acids in the N-terminal region (del75-S271D/T275D), which lacks one of three D domains in MAP2K7 that contribute to the docking site for the substrate JNK[30]. MAP2K7 proteins were incubated with increasing concentrations of ASC69, and kinase activity was quantified as described in Materials and Methods (Fig. 4a). ASC69 inhibited the kinase activity of all three MAP2K7 variants, albeit with different IC_50_ values, indicating that its inhibitory effect is not restricted to any particular conformational state. This is consistent with previous studies showing that ASC69 binds to the ATP-binding domain of MAP2K7[26]. Although the MAP2K7 del75-S271D/T275D mutant was described as an activated state[26], ASC69 inhibitory activity was highest against the wild-type protein and lowest for the phosphomimetic S271D/T275D mutant. These results indicate that ASC69 can inhibit the kinase activity of different MAP2K7 conformations.

To further establish MAP2K7 as a key functional mediator of ASC69 cytotoxicity, we compared ASC69 sensitivity in parental Jurkat cells and CRISPR/Cas9-generated MAP2K7-knockout cells. MAP2K7 loss significantly decreased sensitivity to ASC69, shifting the IC_50_ from 15.0 nM in parental cells to 67.6 nM in MAP2K7-knockout cells (Fig. 4b). Although ASC69 retained measurable cytotoxic activity at higher concentrations in the absence of MAP2K7, the approximately 4.5-fold reduction in sensitivity indicates that MAP2K7 inhibition is a major contributor to ASC69-induced cytotoxicity while suggesting the involvement of additional targets or drug toxicity.

### ASC69 inhibits the downstream JNK-ATF2 pathway

Immunoblot analysis of the MAP2K7 pathway was performed on a panel of T-ALL cell lines to compare vehicle-treated and ASC69-treated cells. ASC69 treatment led to a significant reduction in JNK phosphorylation compared to vehicle controls in T-ALL cell lines, while total JNK expression remained largely unchanged (Fig. 4c), indicating effective suppression of MAP2K7 since JNK is its only substrate. Phosphorylation of the downstream JNK target, ATF2, was significantly decreased in T-ALL cell lines, with some showing reduced total ATF2 protein levels, suggesting that ASC69 may also inhibit ATF2 expression in addition to its activation. These data support the conclusion that ASC69 inhibits JNK in unmanipulated T-ALL cell lines.

To further assess whether ASC69 can inhibit the JNK pathway, KOPT-K1, RPMI-8402, and Ichikawa cells were first treated with sorbitol to induce MAP2K7 activation via osmotic stress, then with ASC69. Sorbitol treatment strongly induced JNK phosphorylation across all three cell lines, confirming effective activation of the stress-induced JNK pathway (Fig. 4d). Subsequent treatment with ASC69 resulted in a dramatic reduction of phosphorylated JNK levels in sorbitol-stimulated cells, while total JNK expression remained unchanged. Analysis of downstream signaling showed that phosphorylated and total ATF2 expression are consistent with those seen under unstimulated conditions (Fig. 4c). These results demonstrate that ASC69 effectively suppresses both basal and stress-induced JNK signaling in T-ALL cells, thereby strengthening its role as a potent inhibitor of MAP2K7-dependent JNK pathway activation under both steady-state and stress conditions.

Given the structural homology between MAP2K7 and MAP2K4 and their shared ability to activate JNK, we also assessed P38 MAPK signaling by measuring phosphorylated and total P38. Interestingly, ASC69 treatment resulted in a pronounced reduction in phosphorylated p38 levels across all T-ALL cell lines examined, while total p38 expression remained unchanged (Fig. 4c). To test whether ASC69 may inhibit MAP2K4, we next investigated whether MAP2K4 inhibition contributes to the reduction in leukemic cell viability. Jurkat cells were treated with the inhibitors DK2403 and GMC09, which are selective for MAP2K7 and MAP2K4, respectively[31]. Cell viability assays showed that ASC69 and DK2403 induced cytotoxicity, whereas GMC09 was less potent, suggesting that cytotoxicity may be driven primarily by MAP2K7 inhibition (Fig. 4e). Immunoblot analysis revealed that ASC69 and DK2403 reduced JNK phosphorylation, whereas ASC69, DK2403, and, to a lesser extent, GMC09 reduced p38 phosphorylation (Fig. 4f). Although GMC09 shows selectivity for MAP2K4 in *in vitro* kinase assays, with IC_50_ values of 1 nM for MAP2K4 and 93 nM for MAP2K7 (not shown), the micromolar IC_50_ in T-ALL cell lines indicates a loss of selectivity at higher concentrations. Collectively, these findings support the conclusion that ASC69-induced inhibition of MAP2K7 predominantly drives cytotoxicity in T-ALL cells.

### ASC69 drug combination and cytotoxicity in human T-ALL cells

We evaluated potential interactions between ASC69 and drugs used in standard-of-care T-ALL treatment (e.g., asparaginase, dexamethasone, and vincristine). We also tested nelarabine and cytarabine, which are commonly used to treat patients with relapsed or refractory leukemia[32, 33]. Analysis of these combination treatments using the Highest Single Agent (HSA) model revealed a dose-dependent synergistic interaction between ASC69 and each therapeutic agent tested (Fig. 5a). Notably, the strongest synergistic effects were observed in Jurkat cells when ASC69 concentrations were near the IC_50_, and when treatment drugs were used at concentrations exceeding their IC_50_ values. These findings suggest that ASC69 and remission-induction drugs activate distinct yet synergistic pathways that induce leukemic cell death. Importantly, the lack of antagonistic interactions across the tested concentrations indicates that suboptimal dosing of either agent is unlikely to decrease therapeutic effectiveness compared to monotherapy.

Next, we evaluated the cytotoxic activity of ASC69 in T-ALL patient-derived xenograft (PDX) cells. Cell viability was assessed using the same ATP-based assay used for T-ALL cell lines; however, due to the limited *ex vivo* viability of primary PDX cells, drug exposure was limited to 24 hours. ASC69 induced dose-dependent cytotoxicity in three independent T-ALL PDX models, with IC_50_ values of 50 nM (relapse T-ALL PDX02), 13 nM (relapse T-ALL PDX03), and 2.5 nM (remission T-ALL PDX04) (Fig. 5b). To assess pathway engagement in PDX cells, immunoblot analysis was performed on treated cells, probing for phosphorylated and total JNK, ATF2, and p38 (Fig. 5c). ASC69 treatment resulted in a pronounced reduction in phosphorylated JNK and ATF2 levels, confirming effective suppression of the MAP2K7-JNK pathway. Notably, lower levels of phosphorylated p38 were detected in ASC69-treated PDX cells relative to untreated controls (Fig. 5c). Finally, ASC69 treatment increased the percentage of Annexin V-positive cells (Fig. 5d).

## Discussion

Pediatric T-ALL remains a hematological malignancy that is difficult to treat, particularly in relapsed and refractory cases, where outcomes remain poor despite intensive multi-agent chemotherapy. Abnormal activation of the stress kinase MAP2K7, driven by epigenetic silencing of KLF4, has emerged as a potential therapeutic target. Our group has reported the anti-leukemic effects of various kinase inhibitors, including JNK-IN-8, 5Z-7-Oxozeaenol, OTSSP167, and DK-2403 [15, 23, 28, 31]. In this study, we demonstrate that ASC69 potently inhibits MAP2K7 in T-ALL models and elicits strong anti-leukemic effects by inducing cell-cycle arrest and apoptosis.

Although ASC69 was initially identified as a small-molecule ligand and a potent inhibitor of the PD-1/PD-L1 interaction [25, 34], to our knowledge, no subsequent studies have evaluated its use as an immune checkpoint inhibitor. Structural and biochemical studies have since shown that ASC69 also binds the active site of MAP2K7 and inhibits its kinase activity [26]. We report here that ASC69 induced cytotoxicity in a panel of T-ALL cell lines at nanomolar concentrations. The role of MAP2K7 in ASC69-mediated cytotoxicity was confirmed in MAP2K7-knockout Jurkat cells, which exhibited reduced sensitivity to ASC69, indicating that the cytotoxic effects largely depend on MAP2K7 inhibition. Mechanistically, ASC69 induces a G2/M cell cycle arrest and apoptosis, consistent with established roles of JNK signaling in regulating mitotic entry, spindle checkpoint integrity, and stress-responsive cell cycle control [35–37]. Similarly, the covalent inhibitor 5Z-7-oxozeaenol (5Z7O) impairs the G2/M checkpoint by downregulating Cyclin B1, CDK1/CDC2, and CDC25C, induces DNA damage, accumulates cells in G_0_, and triggers apoptosis through caspase-3/7 and PARP cleavage in multiple T-ALL lines [23]. Likewise, OTSSP167 induces G2/M arrest and apoptosis in T-ALL cell lines and T-ALL PDX cells, synergizing with standard chemotherapeutic agents [28]. A novel class of covalent MAP2K7 inhibitors, DK-2403, shows promising inhibition of the JNK pathway and cell viability [31].

ASC69 was also shown to inhibit the kinase activity of MAP2K7 conformational mutants, including wild type, phosphomimetic, and truncated forms. The MAP2K7 phosphomimetic mutant that lacks 75 amino acids from the N-terminal region containing the JNK docking domain (D1) is reported to exhibit enhanced catalytic activity due to relief of autoinhibitory constraints [38]. The ability of ASC69 to inhibit this active form of MAP2K7 suggests that its inhibitory activity may be independent of conformational or activation states. Pathway-engagement studies demonstrated that ASC69 treatment markedly reduced JNK phosphorylation under both basal and stress-induced conditions. The downstream effector ATF2 can be activated by JNK and p38, integrating context-specific transcriptional and signaling networks [39, 40]. Given the structural homology between MAP2K7 and MAP2K4, as well as their shared downstream substrate JNK [31], additional biochemical and genetic studies will be needed to distinguish their respective contributions. Nonetheless, these findings suggest that ASC69 may broadly disrupt stress-adaptive MAPK signaling networks that T-ALL cells co-opt to promote survival and therapy resistance. To further support the translational relevance of MAP2K7 inhibition by ASC69, we showed that ASC69 induces dose-dependent cytotoxicity in relapsed T-ALL PDX cells by inhibiting JNK and p38 signaling. Importantly, ASC69 synergizes with vincristine, dexamethasone, asparaginase, nelarabine, and cytarabine, supporting the concept that MAP2K7 inhibitors could be incorporated into existing multidrug treatment regimens rather than administered as standalone agents [41].

In summary, our findings identify ASC69 as a potent inhibitor of MAP2K7-dependent signaling and demonstrate significant antileukemic activity in T-ALL models. By disrupting the stress-activated MAP2K7 pathway, which is critical for cell-cycle progression and survival, ASC69 induces G2/M arrest and apoptosis while selectively targeting T-ALL cells. Future studies should evaluate *in vivo* efficacy, pharmacokinetics, toxicity, and medicinal chemistry optimization to enhance selectivity and translational potential. Overall, our study reinforces MAP2K7 as a therapeutic target in T-ALL and supports the further development of MAP2K7 inhibitors.

## Figures and Tables

**Figure 1:**
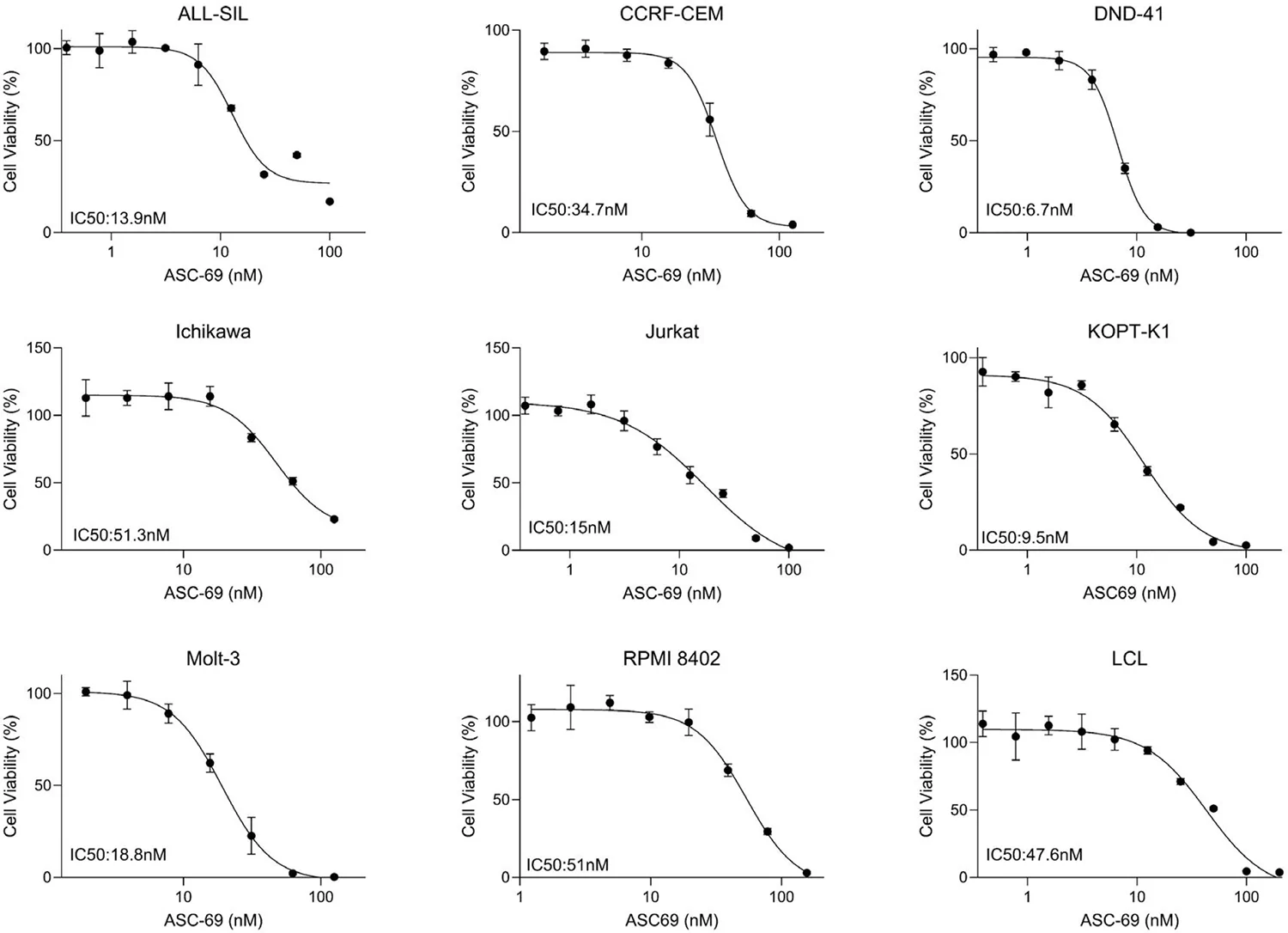
ASC69 inhibits cell viability in T-ALL cell lines. A panel of T-ALL cell lines and LCL cells used as non-leukemic control were treated with different ASC69 concentrations for 48 hours, and cell viability was measured using an ATP-based cell viability assay and expressed as a percentage of vehicle control. Representative cell viability assays of three independent experiments.

**Figure 2:**
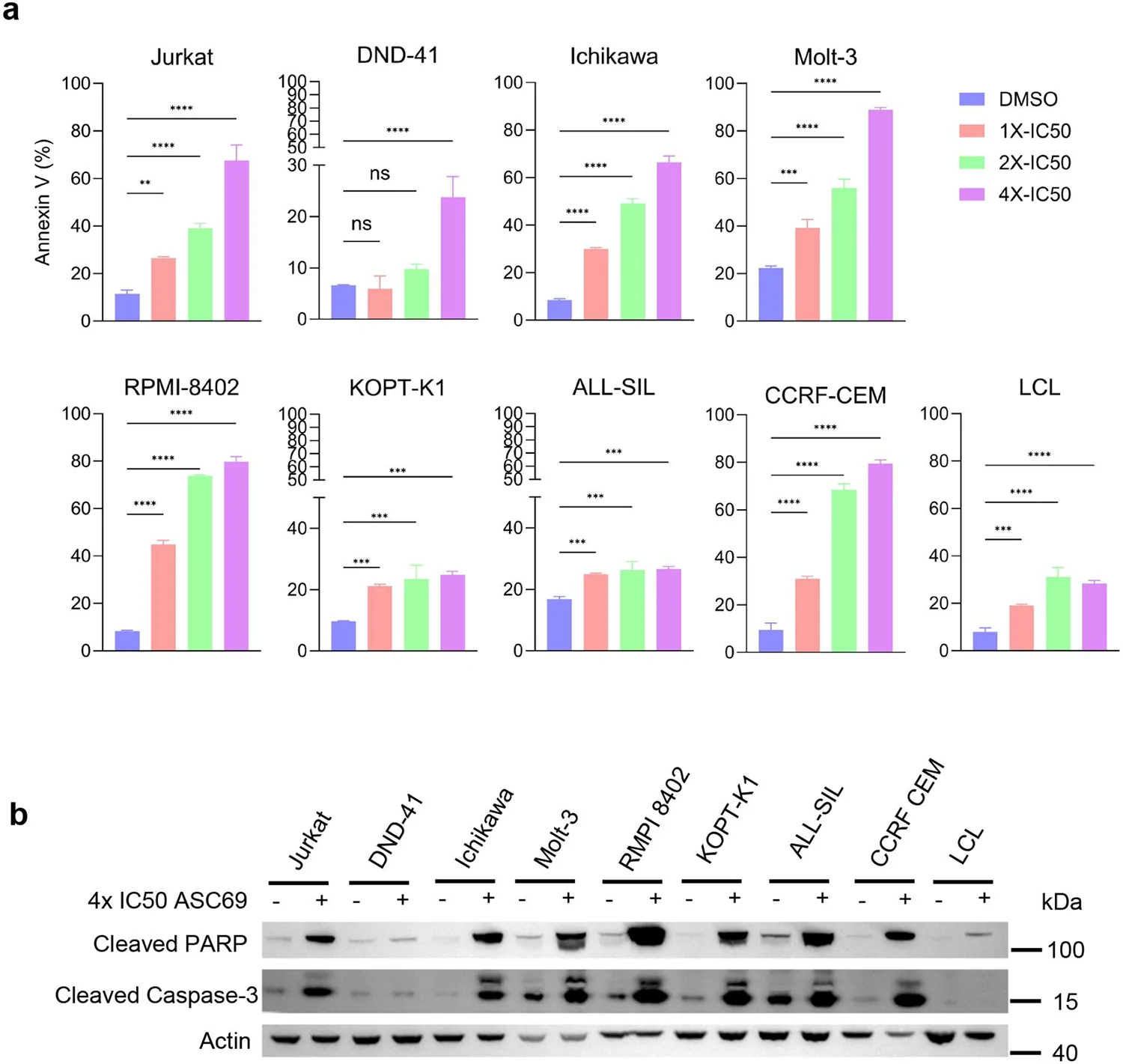
Treatment of T-ALL cells with ASC69 induces apoptosis. (a) Annexin V flow cytometric analysis of T-ALL cell lines treated with three concentrations of ASC69 (1×, 2×, and 4×IC_50_) for 24 hours (n = 3). (b) Immunoblot analysis of cleavage of PARP and Caspase-3. The data represent the mean and standard deviation. NS: no statistical difference. **P<0.01, ***P<0.001, ****P<0.0001 (One-way ANOVA test).

**Figure 3:**
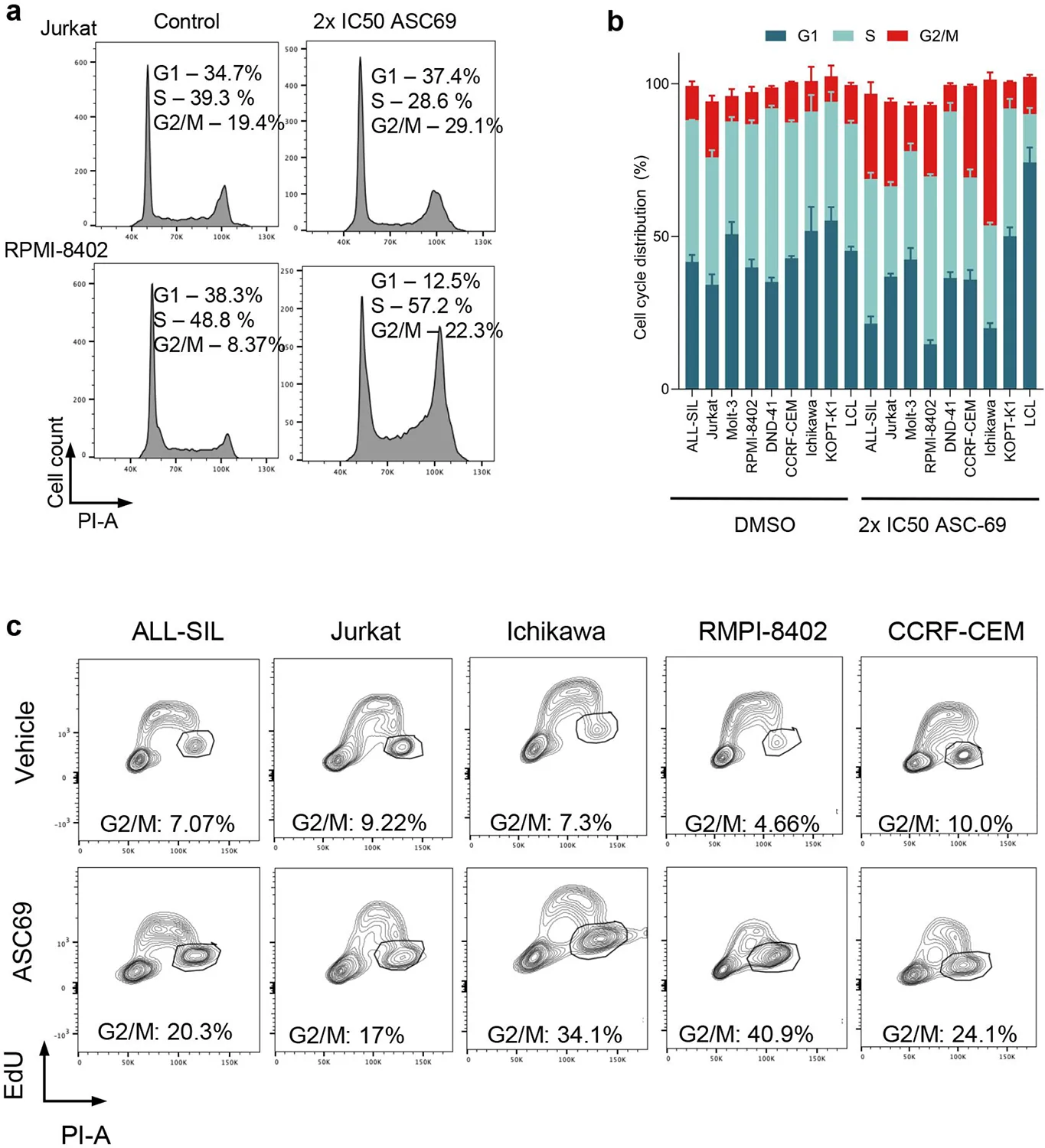
ASC69 induces G2/M cell cycle arrest in T-ALL cell lines. (a) Flow cytometry analysis of DNA content of Jurkat and RPMI-8402 treated with vehicle (DMSO) or 2× IC_50_ ASC69 for 24-hours. (b) Cell cycle distribution of a panel of T-ALL cell lines and LCL treated with vehicle (DMSO) or 2× IC_50_ ASC69 treatment for 24-hours (n = 3). (c) Flow cytometry analysis of cell cycle distribution using EdU and PI in T-ALL cell lines.

**Figure 4:**
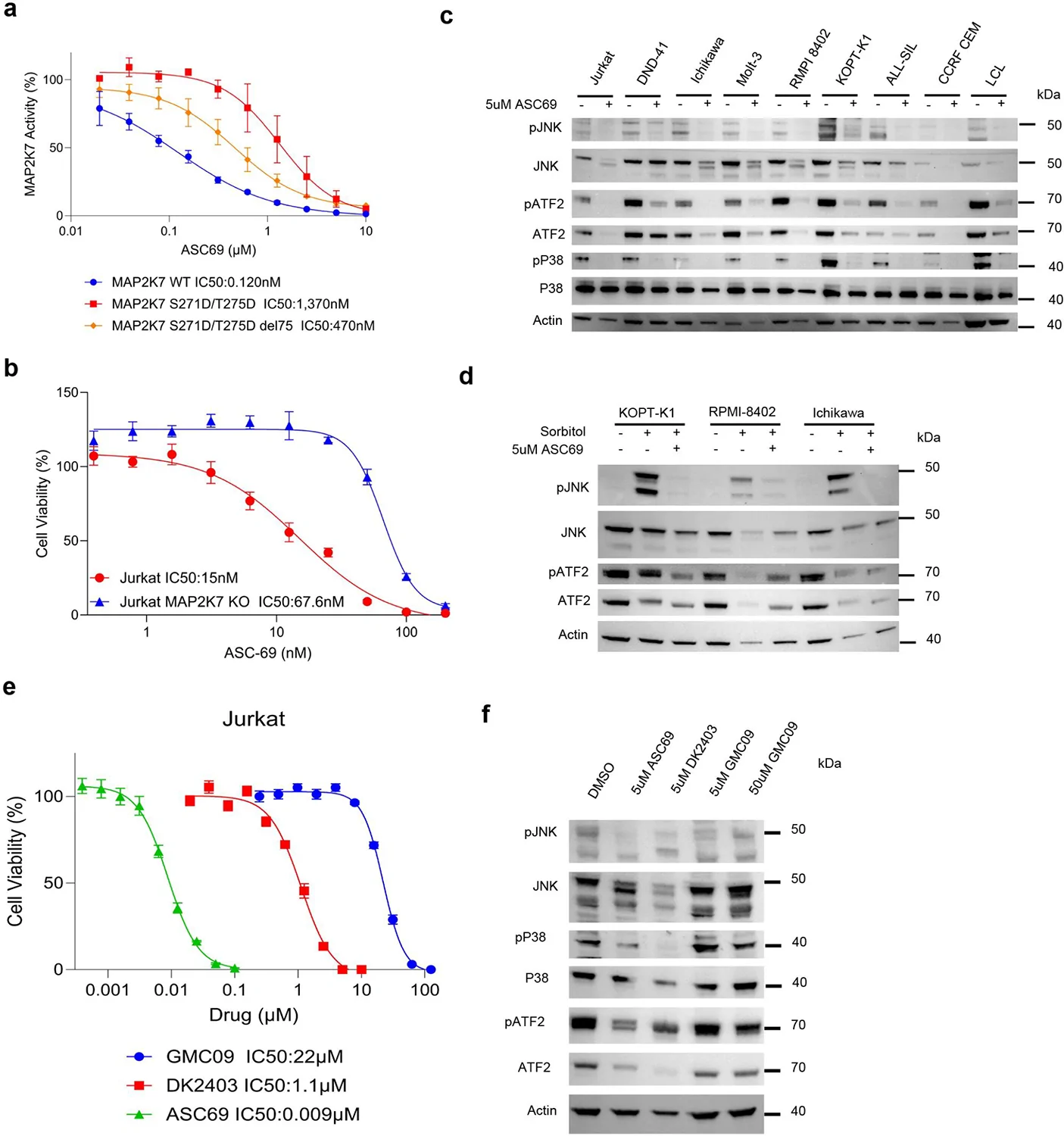
ASC69 inhibits MAP2K7 kinase activity and the downstream JNK/AFT2 pathway. (a) Kinase activity of ASC69 with three variants of human MAP2K7 protein; wild-type (WT), phosphomimetic S271D/T275D and del75-S271D/T275D (n = 3). (b) Cell viability assay of Jurkat and Jurkat MAP2K7 KO cells treated with different concentrations of ASC69 for 48 hours, and cell viability was measured using an ATP-based cell viability assay and expressed as a percentage of the vehicle control (n = 3). (c) Immunoblot analysis of JNK, ATF2, and P38 pathways in a panel of T-ALL cell lines and LCL cells treated with vehicle (DMSO) or ASC69 (5μM for 24hrs). (d) Immunoblot analysis of JNK and ATF2 pathways in T-ALL cells (KOPT-K1, RPMI-8402, Ichikawa) in sorbitol-induced MAP2K7 activation treated with vehicle (DMSO), sorbitol (400 mM), or sorbitol and ASC69. (e) Cell viability assay of Jurkat cells treated with the chemical compounds GMC09, ASC69, and DK2403 for 48 hours, expressed as a percentage of the vehicle control (n=3). (f) Immunoblot analysis of JNK, ATF2 and P38 pathways in Jurkat treated with vehicle (DMSO), ASC69, GMC09 or DK2403 (5μM of each compound and 50μM of GMC09 for 24 hours).

**Figure 5:**
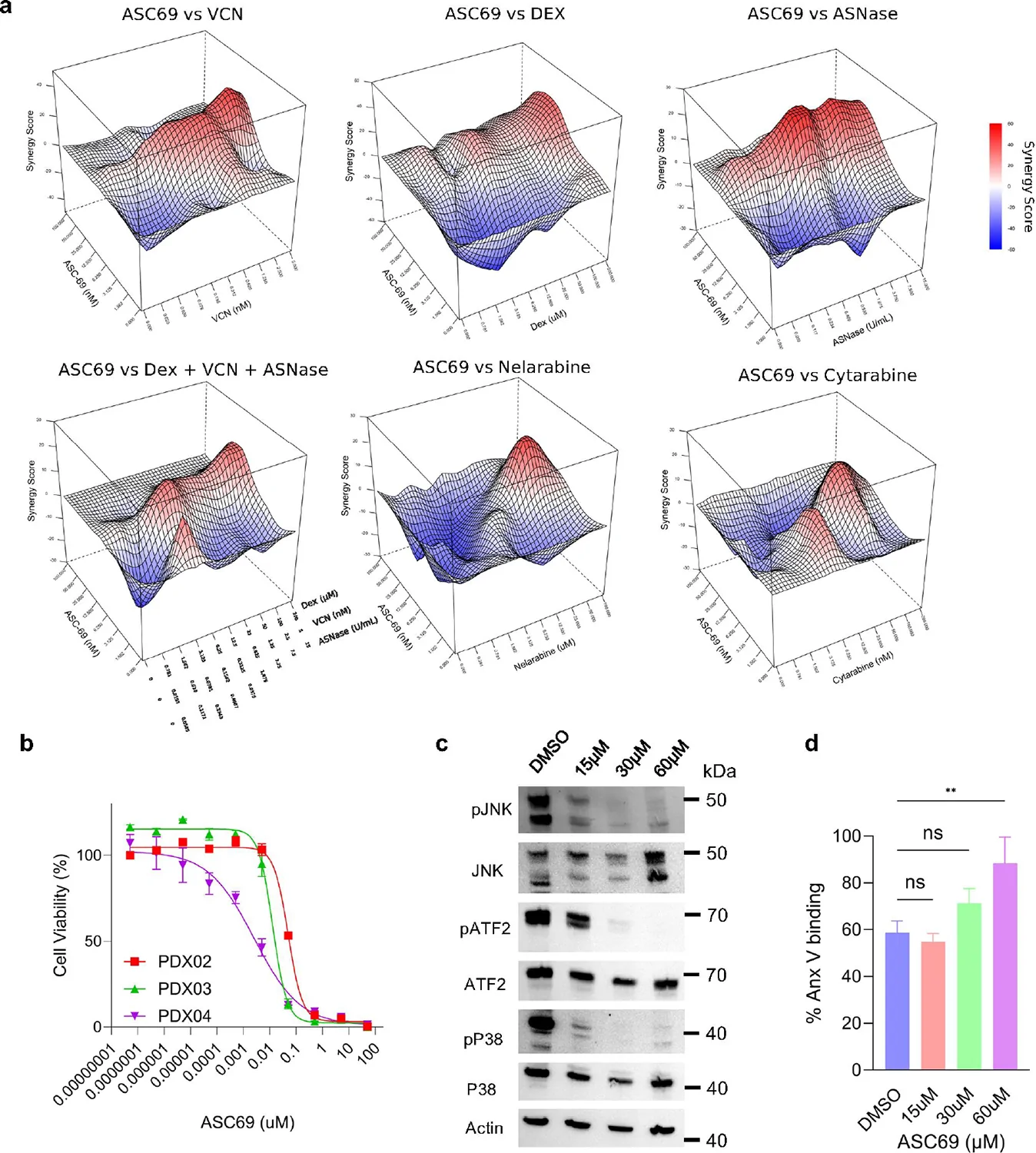
ASC69 drug combination in Jurkat cells and cytotoxicity in relapse T-ALL. (a) Synergy analysis of ASC69 with Vincristine (VCN), Dexamethasone (Dex), Asparginase (ASNase), Nelarabine, and cytarabine in Jurkat cells. Pairwise drug interaction scores were calculated using the highest single agent (HSA) reference model. (b) Cell viability assay of T-ALL PDX cells derived from relapsed (PDX02, PDX03) and remission (PDX04) patients treated with ASC69 for 24 hours. Cell viability was measured using an ATP-based assay and expressed as a percentage of the vehicle control. (c) Immunoblot analysis of JNK, ATF2, and P38 in human PDX T-ALL treated with vehicle (DMSO) or with three concentrations of ASC69 (1×, 2×, and 4× IC_50_) for 24 hours. (d) Annexin V flow cytometry of human T-ALL PDX treated with three concentrations of ASC69 (1×, 2×, and 4× IC_50_) for 24 hours (n = 3). **P<0.01 (One-way ANOVA test).

**Table 1:** ASC69 IC50 for T-ALL cell lines

Cell Line	IC50 48hrs (nM)
KOPT-K1	8.8 ± 0.6
RPMI-8402	52.8 ± 10.0
ALL-SIL	14.2 ± 1.5
Ichikawa	40.7 ± 9.2
CCRF-CEM	32.4 ± 2
Molt-3	20.8 ± 1.8
DND-41	6 ± 0.6
Jurkat	15.3 ± 4.4
Jurkat MAP2K7 KO	67.6 ± 1.4
LCL	47.6 ± 4.4
